# LST-BEV: Generating a Long-Term Spatial–Temporal Bird’s-Eye-View Feature for Multi-View 3D Object Detection

**DOI:** 10.3390/s25134040

**Published:** 2025-06-28

**Authors:** Qijun Feng, Chunyang Zhao, Pengfei Liu, Zhichao Zhang, Yue Jin, Wanglin Tian

**Affiliations:** 1 School of Information Science and Engineering, Shenyang Ligong University, Shenyang 110159, China; 2303620901@stu.sylu.edu.cn (Q.F.); 2403620351@stu.sylu.edu.cn (Y.J.); 2403620339@stu.sylu.edu.cn (W.T.); 2 Shenyang Institute of Automation Chinese Academy of Sciences, Shenyang 110169, China; liupengfei@sia.cn; 3 School of International Studies, Northeast Normal University, Changchun 130024, China; zhangzhichao311@nenu.edu.cn

**Keywords:** autonomous driving, bird’s-eye view (BEV), 3D object detection, large kernel convolution, long-term temporal features

## Abstract

This paper presents a novel multi-view 3D object detection framework, Long-Term Spatial–Temporal Bird’s-Eye View (LST-BEV), designed to improve performance in autonomous driving. Traditional 3D detection relies on sensors like LiDAR, but visual perception using multi-camera systems is emerging as a more cost-effective solution. Existing methods struggle with capturing long-range dependencies and cross-task information due to limitations in attention mechanisms. To address this, we propose a Long-Range Cross-Task Detection Head (LRCH) to capture these dependencies and integrate cross-task information for accurate predictions. Additionally, we introduce the Long-Term Temporal Perception Module (LTPM), which efficiently extracts temporal features by combining Mamba and linear attention, overcoming challenges in temporal frame extraction. Experimental results in the nuScenes dataset demonstrate that our proposed LST-BEV outperforms its baseline (SA-BEVPool) by 2.1% mAP and 2.7% NDS, indicating a significant performance improvement.

## 1. Introduction

With the rapid development of autonomous driving and intelligent transportation systems, 3D object detection, as one of the core tasks in environmental perception, plays a crucial role in safe driving, path planning, and decision control. Traditional 3D object detection methods typically rely on sensors such as LiDAR [1] and radar to acquire spatial depth information [2,3]. However, these sensors are often costly and may underperform in complex scenarios. Therefore, achieving efficient and accurate 3D object detection using only cameras has become an important research direction in visual perception [4].

In purely visual 3D object detection, depth and semantic features are critical elements. Depth features, representing spatial geometric information extracted from images, reflect objects’ shape, position, and relative distance to the camera. While depth features are essential for 3D localization, geometric information alone often lacks semantic understanding, making it difficult to distinguish between different object categories or scene elements accurately. On the other hand, semantic features represent the category, structure, and contextual information of objects in the image, helping the detection system recognize the type of objects and their contextual relationships within the scene. Therefore, effectively fusing depth and semantic features is key to improving the performance of 3D object detection.

Existing methods that primarily utilize depth features can be divided into two categories: explicit extraction methods, represented by [5,6,7,8,9], and transformer-based methods, defined by [10,11,12]. Explicit extraction methods model images’ depth distribution by predicting each pixel’s depth information, allowing a more accurate estimation of the scene’s geometric structure. However, these methods often overlook the importance of semantic information in object detection tasks, failing to fuse depth distribution with semantic information, which limits feature utilization and detection accuracy. On the other hand, transformer-based methods embed 3D information into the model through positional encoding without explicitly predicting depth information, simplifying the model structure. However, the high memory consumption due to the quadratic complexity of transformers remains unresolved, and the lack of explicit depth prediction results in lower accuracy for long-range object detection. Additionally, constrained by traditional convolutional attention mechanisms, existing methods cannot fully exploit long-range dependencies and cross-feature information in images, limiting the extraction of high-precision features.

Meanwhile, transformers have undeniable advantages in handling temporal issues in 3D object detection tasks. Unlike traditional convolutional neural networks (CNNs), self-attention mechanisms can effectively capture global dependencies in images, and temporal attention modules such as [13] can efficiently process temporal information in dynamic scenes, giving them unique advantages in complex tasks like multi-view fusion and cross-frame object tracking. However, challenges such as high computational complexity, insufficient spatial local feature extraction, and limited modeling capability for long-term temporal information remain significant obstacles in current applications.

In recent years, architectures based on state space models (SSMs) [14] have provided new insights into addressing these issues. Compared to the quadratic complexity of traditional transformer models, ref. [14] reduces computational complexity to O(N) through structured state space and selective scanning mechanisms, enabling lower-cost processing of ultra-long sequences (e.g., tens of thousands of tokens). This characteristic offers significant advantages in tasks requiring long-term historical memory. However, since [14] is primarily designed for sequence modeling, it lacks explicit modeling capabilities for spatial local correlations and spatial positional information. Although images can be flattened into sequences for processing [15], this operation disrupts the spatial structure of images, making it difficult for the model to capture local features and geometric structures effectively.

We adopt the convolutional layer design from [16] while innovatively incorporating a feature scaling function layer and proposing the Long-Range Attention (LRSA) module to better adapt to 3D object detection tasks. Furthermore, we connect multiple LRSAs in series to achieve optimal feature extraction performance. By combining LRSA with a cross-task detection head, we propose the Local Spatial Cross-task Detection Head (LSCH) architecture to replace conventional feature extraction tasks. LSCH integrates large-kernel attention mechanisms and cross-task detection methods, utilizing multi-scale large-kernel attention mechanisms to extract image information at different scales, thereby capturing detailed and global information in images while fully exploiting long-range dependencies to establish relationships between distant pixels. This aids in understanding the overall semantics of complex images. Ultimately, multi-task distillation generates depth predictions and semantic features carrying cross-task information. Additionally, this paper proposes a Long-Temporal Perception Module (LTPM) to extract and fuse temporal details in images. Drawing inspiration from the module architecture of [17], we innovatively adapt this framework to the domain of 3D object detection by integrating conventional linear attention mechanisms with Mamba. Considering that recursive computation of state matrices is not ideal for visual models, we use positional encoding to replace recursive computation, allowing it to function as a state matrix in visual tasks. This achieves the goal of adapting to non-causal data like images while extracting long-range spatiotemporal features.

Specifically, our contributions include the following: (1) We propose the Long-Range Cross-task Detection Head (LSCH), which combines multi-scale large-kernel convolution and cross-task information to optimize the perception of depth and semantic information in object detection tasks. (2) We propose the Long-Temporal Perception Module (LTPM), which effectively extracts and utilizes long-term temporal features in images by analyzing and combining the advantages of linear attention and Mamba, addressing the limitations of existing methods in short-term temporal feature extraction. (3) We integrate the above modules with a baseline to form a novel end-to-end 3D object detection framework, LST-BEV. Our experiments demonstrate that LST-BEV achieves a 2.1% improvement in mAP and a 2.7% improvement in NDS on the nuScenes validation dataset compared to the baseline.

This paper is structured as follows: Section 1 states the research objectives and briefly outlines our contributions and achievements. Section 2 reviews related work in the relevant research, covering three key areas: Vision-Based 3D Object Detection, The Image Super-Resolution Task, and Linear Attention Mechanism. Section 3 describes our proposed methods—the Long-Range Cross-Task Detection Head (LSCH) and Long-Term Temporal Perception Module (LTPM). Section 4 presents the datasets and experimental results used for training and comparison, including multiple comparative experiments and ablation studies, demonstrating our proposed model’s advantages. Finally, Section 5 concludes our work with a comprehensive discussion and analysis of the limitations. We have included Appendix A at the end of the article to explain the abbreviations and metrics used throughout the paper.

## 2. Related Work

### 2.1. Vision-Based 3D Object Detection

In early vision-based 3D object detection methods, the most common approach was to extract features from 2D images and directly predict the attributes of 3D objects, such as position, size, and orientation. These methods typically relied on traditional convolutional neural network (CNN) architectures for object detection and obtained 3D information through regression or classification. Ref. [18] is a 2D object detection method, but with minor modifications, it can be extended to 3D object detection. This method first represents objects using center points and estimates their 3D position and size through regression. The advantage of [18] lies in its simplicity and efficiency, making it particularly suitable for real-time applications, and it can perform 3D object detection without additional hardware. Ref. [19] builds upon traditional 2D center point detection by introducing 3D information inference. It detects the 2D center of objects and uses contextual features around the center to infer 3D attributes such as depth, scale, and orientation. The main advantages of these methods are their simple architecture, low computational complexity, and ability to process image data quickly, making them suitable for real-time scenarios. However, their drawbacks are also evident. Due to their reliance on single 2D image features, these methods cannot directly obtain depth information, often resulting in significant errors in 3D object localization.

As research progressed, vision-based 3D object detection methods gradually incorporated more geometric information to improve the accuracy of depth estimation and 3D localization. Ref. [20] is a representative example of such methods. Ref. [20] enhances depth estimation accuracy by constructing geometric relationship graphs. Through multi-stage geometric decomposition, it extracts and fuses geometric information from image features at different scales, thereby improving the precision of 3D object detection. However, geometric enhancement methods like [20] also have certain limitations. First, although they improve the depth estimation accuracy, their high computational complexity leads to slower processing speeds. Additionally, introducing geometric information makes the models more complex, requiring more computational resources and time for training and inference. In some real-time application scenarios, these methods may struggle to meet requirements.

With the increasing maturity of transformer architectures in computer vision [21,22], transformer-based 3D object detection methods have become a research hotspot. Ref. [23] is a representative work in this direction, combining the global attention mechanism of transformers to capture spatial relationships between objects worldwide, thereby achieving more accurate 3D object detection. The strength of [23] lies in its powerful global modeling capability, effectively fusing geometric relationships and semantic information between objects through the transformer structure, thus improving the robustness of 3D object detection. Additionally, ref. [11] introduces 3D position-aware representations to enhance object localization accuracy, especially in complex environments. Ref. [24] incorporates temporal information by leveraging features from consecutive frames to improve detection accuracy and efficiency. Despite their excellent performance in accuracy, transformer-based 3D object detection methods suffer from high computational complexity, particularly in training and inference on large-scale datasets. Moreover, the transformer structure itself demands significant computational resources, which may be challenging to meet in scenarios with high real-time requirements.

In recent years, some methods have proposed frameworks for 3D object detection by transforming image features into bird’s-eye view (BEV) features. BEV provides a top-down perspective, effectively focusing on objects’ spatial layout and position on a plane, making 3D object detection more intuitive and concise. Methods such as [5,8] adopt this BEV-based detection approach by transforming image features into a bird’s-eye view and then predicting 3D objects in this perspective. The main advantage of BEV methods lies in their ability to effectively integrate multi-view information on a 2D plane, reducing reliance on depth estimation and achieving a balance between computational efficiency and detection accuracy. For example, ref. [8] combines the CenterPoint detection head with BEV features to enable fast and accurate 3D object detection. Furthermore, BEV methods are simpler and more extensible than traditional detection methods, requiring no complex post-processing steps. In addition, the combination of BEV features and transformers has been widely applied in the fields of object detection and semantic segmentation. Ref. [25] follows the encoder design of a transformer and implements an efficient deformable attention unit, achieving perspective transformation from BEV maps to images. On this basis, ref. [26] introduced a novel convolutional–transformer hybrid encoder and a multi-level fusion module, realizing precise semantic segmentation for high-definition maps. However, BEV methods also have limitations. First, although they are computationally efficient, effectively incorporating temporal information and handling dynamic object behavior in real-time scenarios remains a challenge. Second, due to their reliance on perspective transformation, BEV methods may encounter perspective issues in specific scenarios (e.g., large-scale open environments), potentially affecting detection accuracy.

### 2.2. The Image Super-Resolution Task

The image super-resolution (SR) task aims to recover high-resolution images from low-resolution counterparts. Over time, SR methods have evolved from traditional interpolation-based approaches to deep-learning-based methods.

Early-image super-resolution techniques mainly relied on interpolation methods for upsampling low-resolution images. Standard interpolation methods include nearest neighbor interpolation, bilinear interpolation, and bicubic interpolation, which perform super-resolution reconstruction by weighting and averaging neighboring pixels. However, these methods show significant limitations in recovering high-frequency details, especially during large-scale upsampling, where the results often exhibit blurring and distortion.

With the rise of deep learning, the super-resolution task has witnessed revolutionary progress. Convolutional neural networks (CNNs) have been widely applied to learn the complex mapping relationship between low-resolution and high-resolution images. Dong et al. (2014) proposed the first end-to-end deep learning super-resolution model [27], significantly improving image reconstruction by learning the nonlinear mapping between low-resolution and high-resolution images. However, due to the shallow architecture of [27], which is incapable of effectively capturing high-frequency details, its performance still had certain limitations. To address this, Kim et al. introduced the [28] model, which incorporated residual learning to enhance feature extraction capabilities, further improving SR performance. Lim et al. proposed [29], which optimized the model structure by removing redundant batch normalization layers, enabling it to learn high-frequency information better. These deep learning-based models made groundbreaking progress in image super-resolution tasks, surpassing traditional interpolation methods and becoming the mainstream approach for SR tasks.

Attention mechanisms and transformer-based models have been another significant development in the super-resolution field in recent years. These methods can better model the global dependencies of images and capture long-range information. Ref. [30] employed a self-attention mechanism to weigh different channels in feature maps, enhancing the focus on critical areas while suppressing the influence of irrelevant regions. This approach effectively improves the recovery of high-frequency details. Furthermore, the Vision Transformer (ViT) has also been applied to super-resolution tasks. Ref. [31] combined the transformer model to capture the global dependencies in images, thereby effectively improving super-resolution performance. Researchers have proposed multi-scale methods to handle scale variations in practical SR tasks better. Ref. [32] introduced a multi-scale method that combines multiple convolutional networks running at different scales, improving super-resolution results for images with highly varied textures. However, the methods above neglect the fixed convolution kernel size, severely limiting the model’s ability to capture long-distance dependencies. Additionally, most existing methods focus on pixel-level reconstruction and overlook the auxiliary role of cross-task features such as semantics and edges in enhancing performance.

Therefore, this paper proposes LSCH, which combines the advantages of multi-scale large convolution and cross-task learning to flexibly address long-distance dependency issues while integrating features from different tasks to optimize image feature perception.

### 2.3. Linear Attention Mechanism

The self-attention mechanism has been a significant breakthrough in deep learning in recent years, particularly in tasks such as natural language processing (NLP) and computer vision (CV). Vaswani et al. [33] proposed that the self-attention mechanism has an O(*N*^2^) computational complexity. This quadratic complexity results in extremely high computational and memory costs when processing long sequences, especially when *N* is large. The linear attention mechanism was introduced to alleviate the computational bottleneck of traditional self-attention. The core idea of linear attention is to reduce the conventional O(*N*^2^) attention computation complexity to O(*N*), significantly improving the efficiency of processing long sequences by using kernel tricks in mathematics. Unlike traditional attention mechanisms, linear attention transforms the interaction between queries and keys into convolution operations, avoiding the dot-product calculation between all positions. This optimization allows linear attention mechanisms to achieve significant computational acceleration while maintaining high representational capability.

The original proposal of linear attention can be traced back to the work of [34], where the dot-product operation in self-attention was replaced with kernel tricks (e.g., using Gaussian kernels) to achieve linear time complexity. This method approximates the interaction between queries and keys through convolution calculations, avoiding explicit computation of the attention matrix, thus significantly reducing computational overhead. The research on linear attention has further developed several variants, including convolution-based attention mechanisms and low-rank decomposition-based attention mechanisms. For example, ref. [35] proposed low-rank self-attention, which reduces computational complexity by utilizing low-rank matrix approximations. At the same time, ref. [29] used a random feature approximation method to calculate the attention matrix, further optimizing the computational complexity of self-attention.

In addition, the linear attention mechanism has been enhanced by introducing techniques such as rotary position encoding (RoPE) and learnable position encoding (LePE), further strengthening the model’s spatial awareness and positional information processing capabilities. However, the aforementioned methods still fail to address the model’s limited ability to capture long-term temporal dependencies. Therefore, this paper proposes the Long-Term Pattern Model (LTPM), which combines the advantages of Mamba and linear attention to extract temporal information from images efficiently.

## 3. Method

**Figure 1:**
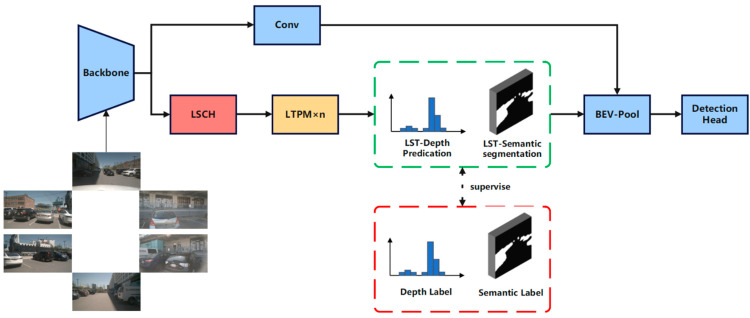
The overall architecture of LST-BEV is as follows. LSCH utilizes long-range attention and cross-scale detection heads to generate depth predictions and semantic segmentation. These are then passed through multiple LTPMs to extract and fuse the temporal features from the depth predictions and semantic segmentation. Finally, after dual supervision, the features are input into the BEV-Pool to generate BEV features.

This section introduces LST-BEV, a novel multi-view 3D object detection framework designed to enhance detection performance by generating high-precision BEV features. It incorporates semantic-aware BEV pooling [36], long-range cross-task detection heads (LSCH), and multiple long-term temporal perception modules (LTPM). Figure 1 illustrates the overall structure of LST-BEV.

LSCH and LTPM will, respectively, extract long spatial dependencies and long temporal dependencies from the image, enhancing the model’s contextual and trend-awareness capabilities. Ultimately, they generate deep predictions with spatiotemporal features and semantic awareness, which are supervised by the projected point cloud on the image through BEVDepth. The depth values of the projected points serve as depth labels, while the points within the 3D detection box represent the foreground. The total loss is as follows:

(1) $$L=L_{\det}+L_{LST-D}+L_{LST-S}$$

Here, *L*_det_ denotes the object detection loss in the detection head, *L_LST-D_* represents the depth label loss, and *L_LST-S_* corresponds to the semantic foreground loss.

BEV-Pool [36] is a semantic-aware pooling layer that performs semantic segmentation based on the semantic information encoded in image features. It computes a foreground score for each feature, where features with lower scores are likely to contain irrelevant information for 3D object detection. During the BEV pooling process, such features are filtered out by discarding their corresponding virtual points. This mechanism ensures that only valuable foreground information and meaningful virtual points are preserved in the final BEV representation. The final depth predictions and semantic awareness are input into the BEV-Pool to generate high-precision BEV feature maps.

### 3.1. Long-Range Cross-Task Detection Head (LSCH)

In autonomous driving scenarios, one of the challenges faced by 3D object detection tasks is the multi-scale nature of targets and complex contextual dependencies. For example, a distant vehicle may occupy a small pixel area, while a nearby pedestrian may occupy a larger pixel area. A large receptive field is required to capture global information to detect these targets, rather than just local features accurately. Traditional convolutional neural networks, due to their fixed receptive fields and small convolution kernels, such as (3 × 3), cannot capture both local details and global context information simultaneously, leading to limited detection accuracy.

Semantic features provide high-level knowledge of 3D objects, such as category information, object properties (e.g., material, shape, etc.), and relationships with the scene. This semantic information can be shared across tasks, helping the model understand the context of objects during detection. However, experiments show that using a single branch for both semantic awareness and depth prediction results in suboptimal solutions for both tasks. Specifically, when the depth branch is used to predict semantic features, the results tend to be suboptimal, reducing the accuracy of BEV feature predictions.

**Figure 2:**
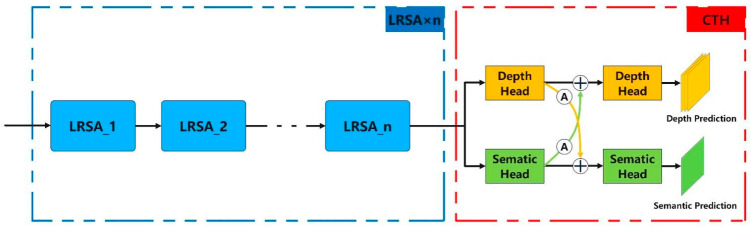
The overall structure of LSCH consists of n LRSAs and 1 CTH, where A represents the cross-task weighted attention and + denotes feature concatenation.

This paper improves multi-scale large-kernel convolutions to address these issues and applies them to the autonomous driving object detection domain. A Long-Range Cross-Task Detection Head (LSCH) is proposed. By leveraging the significant receptive field characteristics of large-kernel convolutions (5 × 5, 7 × 7, 9 × 9), LSCH better captures long-range dependencies and multi-scale features present in the scene while simultaneously generating high-precision depth predictions and semantic information. Specifically, LSCH consists of two main parts: multiple Long-Range Attention (LRSA) and a Cross-Task Detection Head (CTH), with the overall structure shown in Figure 2.

LRSA, as an improved multi-scale large-kernel convolution, effectively enlarges the receptive field and captures long-range dependencies between distant regions in the image while simultaneously handling fine-grained features, alleviating the limitations of small convolution kernels. Furthermore, LRSA can flexibly adjust the size and combination of convolution kernels based on the task. By designing convolution kernels at different scales, the model can adapt to the feature extraction requirements of various tasks, making it an effective method for extracting long-range features. Cross-task detection can significantly enhance the model’s multi-tasking ability, strengthening the synergy between tasks, sharing feature representations, and improving detection accuracy. In object detection tasks, integrating and utilizing both depth predictions and semantic information is crucial for enhancing detection accuracy, improving scene understanding, and adapting to the demands of complex environments. Based on this, the proposed LSCH demonstrates significant advantages in extracting high-precision, long-range information and better meeting the needs of complex scene understanding in real-world applications.

#### 3.1.1. Long-Range Attention (LRSA)

**Figure 3:**
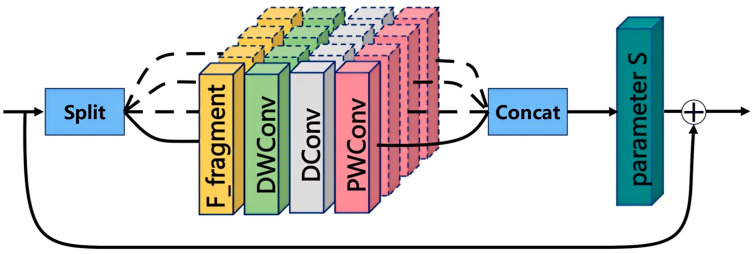
The structure of LRSA, where + denotes feature addition in the residual connection.

To fully leverage the advantages of large-kernel convolutions with large receptive fields while avoiding excessive feature mixing and retaining more local features, thus enhancing multi-scale feature representation, we propose Long-Range Spatial Attention (LRSA). The core idea is to segment the input features along the channel dimension and apply convolution kernels of different sizes to sub-features at various scales, capturing global context while preserving local details. The structure of LRSA is shown in Figure 3.

Specifically, for the input feature map *X = ℝ^C^^×H^^×W^*, LRSA splits the features along the channel dimension into *n* sub-parts, denoted as *x*_1_, *x*_2_, *…*, *x*_3_*,* where each sub-part has a scale of $\frac{C}{n}\times H\times W$. Each sub-part *x_i_* is then input into *n* different large-kernel convolution blocks, denoted as LKA_n_. Each LKA consists of three main components: depthwise convolution (DepthwiseConv, *f_DWC_()*), dilated convolution (DilatedConv, *f_DC_())*, and point-wise convolution (Point-wiseConv, *f_PC_()*), which the following formula can express:

(2) $$LKA(X)=f_{PW}(f_{DC}(f_{DWC}(X)))$$

Specifically, we design four different scales for the LKA: {1, 3, 1}, {3, 5, 1}, {5, 7, 1}, and {7, 9, 1}, where {a, b, c} represent the kernel sizes for depthwise convolution, dilated convolution, and pointwise convolution, respectively. Larger-scale LKAs are effective at capturing long-range dependencies in features, while smaller-scale LKAs are more inclined to preserve local textures and fine details. Additionally, LRSA addresses the vanishing gradient problem in deep network training through residual connections. The final computation formula for LRSA can be expressed as

(3) LRSA(X)=LKA(X)×S+X

Here, *S* denotes the feature scaling function, and *LKA(X) × S* represents the scaled output of the *LKA*.

#### 3.1.2. Cross-Task Detection Head (CTH)

In autonomous driving scenarios, 3D object detection tasks not only require accurate localization of the targets but also need to understand the semantic information of the objects (e.g., category, attributes, etc.) and their contextual relationships with the environment. However, traditional detection methods treat depth prediction and semantic segmentation as completely independent tasks, leading to feature extraction and utilization limitations. Research has shown that task-specific and cross-task information is crucial for obtaining a global optimal solution for multi-task learning. To address this issue, we propose the Cross-Task Detection Head (CTH), which aims to fully utilize depth perception, semantic segmentation information, and the complementary information between them through a multi-task distillation mechanism.

Specifically, the input features *F* pass through detection heads for depth prediction and semantic segmentation, generating features *F_D_* that carry depth information and *F_S_* that carry semantic information, respectively. To achieve cross-task detection, we introduce a multi-task distillation module (MTD), which consists of several cross-attention modules, denoted as Att. Each attention module performs a weighted convolution operation to fuse the features of the two tasks, enabling the transfer and sharing of cross-task information. The attention module can be expressed as:

(4) Att(x×y)=Conv(x)⊙σ(Conv(y))

Here, *Conv*() denotes a 3 × 3 gated convolution operation; ⊙ represents element-wise multiplication; and *σ* refers to the Sigmoid activation function. With this design, the attention module dynamically adjusts feature weights based on the importance of the task, allowing the result to simultaneously incorporate feature information from both *x* and *y*, thereby achieving an effective fusion of cross-task information.

Based on cross-task feature fusion, CTH enables the bidirectional transfer of depth and semantic features through a multi-task distillation module. Specifically, depth feature *F_D_* and semantic feature *F_S_* are weighted and fused through the attention module to generate *F_D_S_* and *F_S_D_*, which carry cross-task information. The calculation formulas are as follows:

(5) $$\left\{\begin{array}{c} F_{D\_S}=F_{D}+(Conv(F_{S})⊙\sigma (Conv(F_{D})))\\ F_{S\_D}=F_{D}+(Conv(F_{D})⊙\sigma (Conv(F_{S}))) \end{array}\right.$$

It is evident that CTH automatically extracts cross-task information from one task’s features using these attention modules and adds it to the features of other tasks, resulting in depth and semantic features that carry cross-task information. The fused cross-task information is then fed into their respective detection heads, which predict high-precision depth distributions and semantic segmentation. These predictions are subsequently used to generate BEV features.

### 3.2. Long-Term Temporal Perception Module (LTPM)

**Figure 4:**
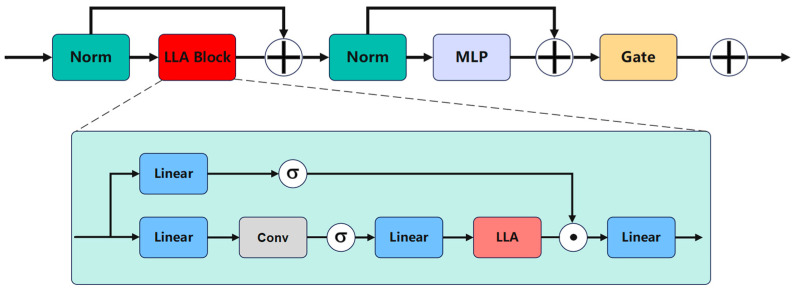
The structure of LTPM, where “·” represents Hadamard Product, and ”σ” represents SiLU Activation.

In 3D object detection, modeling temporal information is crucial for understanding the motion trends of objects in dynamic scenes. Traditional convolutional neural networks (CNNs) and transformers have certain limitations when handling long-term temporal information: CNNs have a limited receptive field, making it challenging to capture long-range dependencies, while transformers, although capable of capturing global information through self-attention mechanisms, suffer from high computational complexity, especially when processing long sequences, resulting in significant memory and computational overhead. To address these issues, we propose the Long-Term Temporal Perception Module (LTPM), which combines the advantages of linear attention mechanisms and the Mamba architecture. This effectively captures long-term temporal features while maintaining linear computational complexity. The structure of LTPM is shown in Figure 4.

Here, Norm denotes the normalization layer, whose primary function is to independently normalize the features of each sample. This operation effectively stabilizes the training process and accelerates convergence.

Traditional linear attention mechanisms reduce the computational complexity of attention calculations to O(*N*) using kernel tricks, enabling efficient processing of long sequence data. The core idea is to transform the interaction between queries and keys into convolution operations, thus avoiding explicit dot-product calculations. The formula is expressed as follows:

(6) $$Q=\phi (xW_{Q});K=\phi (xW_{K});V=xW_{V};y_{i}=\frac{Q_{i}(\sum_{j=1}^{N}K_{j}^{T}V_{j})}{Q_{i}(\sum_{j=1}^{N}K_{j}^{T})}$$

Here, *W_Q_*, *W_K_*, and W_V_ represent the projection matrices, and *Q*, *K*, and *V* represent the query, key, and value matrices, respectively. *Q_i_*, *K_i_*, and *V_i_* denote independent queries, keys, and values, while *ϕ* represents the kernel function. However, linear attention still faces challenges when processing long-term temporal information, such as information loss, position encoding loss, and difficulties in modeling long-range dependencies.

As a variant of linear attention, Mamba employs a structured state space and selective scanning mechanism, which enables it to handle ultra-long sequences with a complexity of O(*N*). The core idea is to recursively model the input sequence using state matrix *A*, thereby capturing long-term temporal dependencies. However, state matrix *A* is highly sensitive to the input sequence and causal data, making it unsuitable for non-causal data such as images. Moreover, the Mamba architecture is primarily designed for sequence modeling tasks, lacking the ability to model spatial local correlations and position information explicitly.

Experimental results show that state matrix *A* provides two key attributes to the model: local bias and position information. This demonstrates that the state matrix *A* in the classical Mamba model can be replaced by an appropriate position encoding. It supplies the model with local bias and position information, making it suitable for non-auto-regressive vision models. Therefore, we propose the Long-Term Temporal Linear Attention (LLA), which can be expressed as:

(7) $$Q=\bar{A}\phi (xW_{Q});K=\bar{A}\phi (xW_{K});V=xW_{V};y_{i}=\frac{Q_{i}(\sum_{j=1}^{N}K_{j}^{T}V_{j})}{Q_{i}(\sum_{j=1}^{N}K_{j}^{T})}$$

Here, $\bar{A}$ represents the position encoding, which is used to provide local bias and position information. Through this approach, LLA is able to effectively capture long-range dependency features while maintaining linear complexity.

Finally, we design a Mamba-like module structure, where the LLA-Block replaces the selective SSM, and the MLP module from the linear attention transformer is added. Based on this, we introduce a learnable gating mechanism (Gate), allowing the model to adjust the strength of its outputs adaptively. This enhancement improves the model’s ability to capture complex temporal features.

## 4. Experiments

This section introduces the dataset and experimental setup used in our study, followed by a comparison with previous state-of-the-art multi-view 3D object detection methods. Finally, we conduct a series of ablation experiments and a comprehensive analysis to demonstrate the effectiveness of various components, namely LSCH and LTPM, in our proposed LST-BEV framework.

### 4.1. Dataset and Metrics

NuScenes is a large-scale autonomous driving benchmark dataset. It consists of 750 scenes for training, 150 for validation, and 150 for testing. Each scene lasts about 20 s, and key samples are annotated at a frequency of 2 Hz. The data for each sample is collected from six cameras, one LiDAR, and five radars. For 3D object detection, the nuScenes detection score (NDS) is introduced to evaluate various aspects of the nuScenes detection task. In addition to the mean average precision (mAP), NDS is also associated with five types of true-positive (TP) metrics, including the mean translation error (mATE), mean scale error (mASE), mean orientation error (mAOE), mean velocity error (mAVE), and mean attribute error (mAAE).

The mAP (mean Average Precision) is used to comprehensively evaluate the model’s detection capabilities across all target categories, including recall and localization accuracy, while the NDS (NuScenes Detection Score) is a weighted composite of mAP and five true-positive errors (mATE, mASE, mAOE, mAVE, and mAAE). Given that the NDS already incorporates all mTP errors and that mAP + NDS can cover over 80% of performance analysis requirements, this paper opts to use mAP + NDS for detailed analysis in select comparative experiments and all ablation studies to highlight the core findings and maintain data conciseness.

### 4.2. Implementation Detail

The BEVPool network structure was improved based on MMDetection3D (MMDetection3D 1.4.0) and two NVIDIA GeForce RTX 4090 GPUs (ASUS Suzhou Factory, Suzhou, Jiangsu, China). The model was trained using the AdamW optimizer with gradient clipping. We used ResNet-50 as the image backbone during training and downsampled the images to a resolution of 256 × 704 as input, training for 24 epochs. The model parameters are as follows: Training time: 42 h 53 min; Number of parameters: 83.29 M; FPS: 9.1 img/s.

### 4.3. Main Result

#### 4.3.1. Comparison with Both Previous Classic Approaches and SOTA Methods

We compared LST-BEV with classic multi-view 3D detection methods on the nuScenes test set, and the results are shown in Table 1. The experimental results in Table 1 demonstrate that our proposed model maintains significant accuracy advantages over conventional baseline methods across different backbone architectures and input image resolutions. However, due to the limited number and quality of GPUs available for training, we were unable to use high-precision backbones, such as V2-99, Swin-B, or ResNet-101, and high-resolution images as inputs, which prevented us from directly comparing our experimental results with existing state-of-the-art (SOTA) methods. Nevertheless, we reproduced several state-of-the-art 3D object detection methods using ResNet-50 as the backbone. We compared them with our proposed method on the nuScenes validation dataset, as shown in Table 2. It can be observed that LST-BEV achieved the best detection results, with a 2.1% improvement in mAP and a 2.7% improvement in NDS over its baseline (i.e., BEVPool), demonstrating its notable potential and advantages. Moreover, even when employing lower input resolutions and a less complex backbone, our model outperforms CAPE by +1.5% mAP and +7.8% NDS and surpasses FCOS3D by +1.9% mAP and +6.9% NDS.

#### 4.3.2. VisualizationBEV

**Figure 5:**
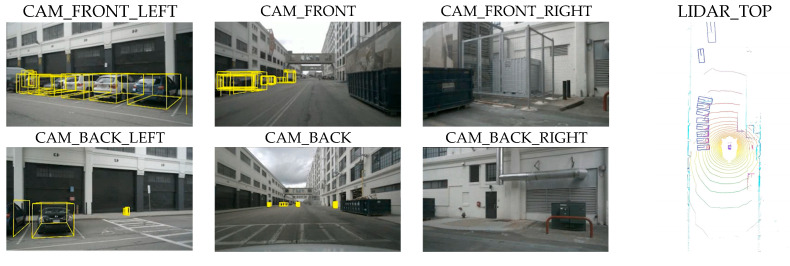
The visualization of LST-BEV detection results on the nuScenes val set.

Figure 5 presents a set of detection results in complex scenarios. The results demonstrate that LST-BEV maintains accurate object classification and precise localization even under challenging conditions with various obstructions such as containers and electrical boxes. This indicates that our model achieves high detection accuracy while exhibiting strong robustness against environmental interference.

### 4.4. Ablation Study

#### 4.4.1. Component Analysis

We used SA-BEVPool as the baseline and evaluated the contributions of LSCH and LTPM, with the results shown in Table 3. After incorporating LSCH, mAP and NDS increased by 1.6% and 1.1%, respectively. After incorporating LTPM, mAP increased by 0.5% and NDS increased by 1.6%. Ultimately, the complete LST-BEV model achieved a total improvement of 2.1% in mAP and 2.7% in NDS, validating the effectiveness of our improvements. We attribute this improvement to LSCH’s ability to extract long-distance features and LTPM’s capability to capture and fuse long-term temporal dependencies. This addresses the limitations of previous object detection methods, which were constrained by local receptive fields and short-term dependencies, thereby enhancing detection accuracy in complex scenes.

#### 4.4.2. Ablation Experiments on the Long-Range Cross-Task Detection Head (LSCH)

The LSCH consists of n Long-Range Spatial Attention (LRSA) modules and one Cross-Task Detection Head (CTH). Through multiple experiments, we further validated the effectiveness of each module and determined the optimal number of LRSA modules. The results are presented in Table 4. Our CTH and a single LRSA module improved the performance of the LSCH by 1.0% mAP and 0.6% NDS, and 0.5% mAP and 0.5% NDS, respectively. The maximum improvement, achieved with two LRSAs, was 0.6% mAP and 0.6% NDS.

We attribute this improvement to the LSCH’s ability to capture long-range spatial features, allowing the model to better capture the targets’ long-distance dependencies. Furthermore, the CTH’s cross-task feature fusion of depth prediction and semantic information enables complementary and synergistic utilization of different features, effectively enhancing detection accuracy.

#### 4.4.3. Ablation Experiments on Long-Term Temporal Perception Module (LTPM)

In this section, we first validate the effectiveness of the Long-Term Perception Module (LTPM) and demonstrate the necessity of gated convolutions. Ultimately, we identify the optimal number of LTPMs, as shown in Table 5. The LTPM without gated convolutions and the LTPM with gated convolutions improve the baseline by 0.1% and 0.3% mAP, respectively. The most significant improvement is achieved when two LTPMs are used, resulting in a 0.5% mAP and a 1.6% NDS increase. Notably, the experimental data indicate that, after employing LTPM, the NDS shows a more pronounced improvement than the mAP. This suggests that LTPM enhances detection accuracy and effectively corrects direction and scale errors. We attribute this improvement to adding long-term sequence features, which enable the model better to understand the target’s motion trends and speed differences. Moreover, these features assist the model in more accurately recognizing scale variations, thereby improving the overall detection performance.

## 5. Conclusions and Discussion

This paper proposes LST-BEV to extract and utilize long-range and long-term sequential features from images. LSCH effectively captures long-range dependencies in the image while extracting cross-task information by combining the improved large-kernel attention LRSA and the cross-task detection head CTH. This leads to the generation of high-precision depth predictions and semantic information with long-range features. LTPM integrates the advantages of linear attention mechanisms and Mamba, utilizing position encoding to replace recursive state matrices, enabling fast temporal information extraction within limited storage space. Experimental results demonstrate that LST-BEV achieves significant improvements over the baseline and exhibits substantial advantages compared to other state-of-the-art models.

Research and experiments demonstrate that effectively extracting and utilizing long-range multimodal cross-task fusion information and long-term temporal features is critical for improving accuracy in 3D object detection tasks for autonomous driving. Experimental results indicate that long-range multi-scale feature extraction and cross-task feature fusion can effectively address the inability to capture long-range dependencies and insufficient feature utilization in object detection, thereby significantly enhancing detection precision. Furthermore, the effective extraction and utilization of long-term temporal information enable the model to better understand and predict object motion trends, improving overall detection performance.

However, it should be noted that, due to limitations in the quantity and quality of GPUs available for training, we were unable to employ high-precision backbone networks or high-resolution images as inputs, resulting in experimental outcomes that cannot be directly compared with state-of-the-art (SOTA) methods. Additionally, since our approach extracts, utilizes, and fuses multiple image features, the model requires increased parameter processing and training time, imposing additional burdens on model instantiation and real-time detection. This remains an area for future research.

## Figures and Tables

**Table 1:** We compared LST-BEV with previous classic multi-view 3D detection methods on the nuScenes test set, where **↑** indicates better performance with increasing values of the metric, while **↓** represents better performance with decreasing values. Our proposed method consistently improves by at least 1.8% mAP and 5.2% NDS compared to the other methods presented in the table.

Method	mAP ↑	NDS ↑	mATE ↓	mASE ↓	mAOE ↓	mAVE ↓	mAAE ↓
FOCSD	0.358	0.448	0.690	0.249	0.452	1.434	0.124
DHNet	0.363	0.437	0.667	0.259	0.402	1.589	0.437
Mono3D	0.366	0.429	0.642	0.252	0.523	1.591	0.119
FCOSD	0.358	0.428	0.690	0.249	0.452	1.434	0.124
CenterNet	0.338	0.400	0.658	0.255	0.629	1.629	0.142
LST-BEV	0.384	0.500	0.619	0.263	0.497	0.383	0.159

**Table 2:** We compared LST-BEV with previous state-of-the-art multi-view 3D detection methods on the nuScenes val set, where **↑** indicates better performance with increasing values of the metric, while **↓** represents better performance with decreasing values. Our proposed method demonstrates significant improvements over the baseline, achieving gains of 2.1% mAP and 2.7% NDS. Furthermore, it outperforms all other comparative methods in the table by substantial margins of at least 0.4% mAP and 4.9% NDS.

Method	Backbone	Resolution	mAP ↑	NDS ↑
FCOS3D	ResNet-101	900 × 1600	0.343	0.415
DETRD	ResNet-101	900 × 1600	0.303	0.374
BEVFormer	ResNet-50	900 × 1600	0.351	0.426
BEVDet	ResNet-50	900 × 1600	0.283	0.350
BEVDepth	ResNet-50	900 × 1600	0.330	0.435
DualBEV	ResNet-50	256 × 704	0.358	0.433
CAPE	ResNet-50	512 × 1408	0.347	0.406
TiG-BEV	ResNet-50	256 × 704	0.338	0.375
SA-BEVPool	ResNet-50	256 × 704	0.341	0.457
Our method	ResNet-50	256 × 704	0.362	0.484

**Table 3:** Ablation experiments on the components of LST-BEV were conducted on the nuScenes validation set, where **↑** indicates better performance with increasing values of the metric, while **↓** represents better performance with decreasing values. The checkmark “√” indicates the names of the added modules.

Baesline(BEV Pool)	LSCH	LTPM	mAP ↑	NDS ↑
			0.341	0.457
Our method	√		0.357	0.468
	√	√	0.362	0.484

**Table 4:** Ablation experiments on the nuScenes val set for LSCH, where CTH and LRSA represent the Cross-Task Detection Head and Long-Range Spatial Attention Module, respectively. “√” indicates the incorporation of the CTH module. “×n” indicates the number of instances used. “↑” indicates better performance with increasing values of the metric, while “↓” represents better performance with decreasing values. The optimal experimental results were achieved when incorporating two LRSA modules.

CTH	LRSA	mAP ↑	NDS ↑
		0.341	0.457
√		0.351	0.463
√	×1	0.356	0.468
√	×2	0.357	0.468
√	×3	0.354	0.467

**Table 5:** We conducted an ablation study of the Long-Term Perception Module (LTPM) on the nuScenes val set, where LTPM- refers to the LTPM without gated convolutions, and ×n denotes the number of LTPMs used. The optimal experimental results were achieved when incorporating two LTPM modules.

Method	mAP	NDS
	0.357	0.468
LTPM-	0.358	0.470
LTPM×1	0.360	0.480
LTPM×2	0.362	0.484
LTPM×3	0.359	0.476

## Data Availability

Data are contained within the article.

## Appendix A

**Abbreviation**	**Full Term**	**Mathematical Formulation/Definition**
BEV	Bird’s-Eye View	
LRSA	Long-Range Attention	
CTH	Cross-Task Detection Head	
LRCH	Long-Range Cross-Task Detection Head	
LTPM	Long-Term Temporal Perception Module	
LST-BEV	Long-term Spatial-Temporal Bird’s-Eye-View	
CNNs	Convolutional Neural Networks	
Norm	Normalization	Norm denotes the normalization layer, whose primary function is to independently normalize the features of each sample. This operationeffectively stabilizes the training process and accelerates convergence.
BEV-Pool	Semantic-aware pooling layer	BEV-Pool is a semantic-aware pooling layer that performs semantic segmentation based on the semantic information encoded in image features. It computes a foreground score for each feature, where features with lower scores are likely to contain irrelevant information for 3D object detection. During the BEV pooling process, such features are filtered out by discarding their corresponding virtual points. This mechanism ensures that only valuable foreground information and meaningful virtual points are preserved in the final BEV representation.The final depth predictions and semantic awareness are input into the BEV-Pool to generate high-precision BEV feature maps.
conv	Convolutional Operation	$\mathrm{conv}\left(i,j\right)=\overset{k_{h}-1}{\underset{m\;=0}{\sum }}\overset{k_{w}-1}{\underset{n\;=0}{\sum }}I\left(i\;+\;m,j\;+\;n\right)\cdot K\left(m,n\right)+\;b$ I:Input feature map - K: k_h_ × k_w_ convolution kernel - B: Bias term
SSMs	State Space Models	
mAP	mean Average Precision	$\mathrm{mAP}\;=\frac{1}{C}\underset{c\in }{\sum }AP_{c}$ *C*: Set of target classes *AP_c_*: Average Precision for class c
NDS	NuScenes Detection Score	$\mathrm{NDS}\;=\frac{1}{2}[mA+\underset{m\in M}{\sum }\left(1-min\left(1,m\right)\right)]$ *M* = {mATE,mASE,mAOE,mAVE,mAAE}M = {mATE,mASE,mAOE,mAVE,mAAE} contains five true-positive error metrics
mATE	Mean Average Translation Error	$\mathrm{mATE}\;=1-\frac{1}{N}\overset{N}{\underset{i=1}{\sum }}{\left|\left|t_{i}^{pred}-t_{i}^{gt}\right|\right|}_{2}$ *t^pred^*: Predicted bounding box center coordinates (x, y, z). *t^gt^*: Ground truth bounding box center coordinates.
mASE	Mean Average Scale Error	$\mathrm{mATE}\;=1-\frac{1}{N}\overset{N}{\underset{i=1}{\sum }}IoU(B_{i}^{pred}\;,B_{i}^{gt})$ *B^pred^*: Predicted bounding box dimensions (length, width, height). *B^gt^*: Ground truth bounding box dimensions.
mAOE	Mean Average Orientation Error	$\mathrm{mAOE}\;=\frac{1}{N}\overset{N}{\underset{i=1}{\sum }}|\theta_{i}^{pred}-\theta_{i}^{gt}|\;\;\;\;\left(\theta \;\epsilon \;\left[-\pi ,\;\pi \right]\right)$ $\theta^{pred}$: Predicted yaw angle. $\theta^{gt}$: Ground truth yaw angle.
mAVE	Mean Average Velocity Error	$\mathrm{mAVE}\;=1-\frac{1}{N}\overset{N}{\underset{i=1}{\sum }}{\left|\left|v_{i}^{pred}-v_{i}^{gt}\right|\right|}_{2}$ v*^pred^*: Predicted velocity vector. v^gt^:Ground truth velocity
mAAE	Mean Average Attribute Error	$\mathrm{mAAE}\;=\frac{1}{N}\overset{N}{\underset{i=1}{\sum }}Ⅱ(a_{i}^{pred}\;\neq \;a_{i}^{gt})$ *a^pred^*: Predicted attribute *a^gt^*: Ground truth attribute II(): Indicator function
